# Lung Cancer and Interstitial Lung Diseases: A Systematic Review

**DOI:** 10.1155/2012/315918

**Published:** 2012-07-29

**Authors:** Kostas Archontogeorgis, Paschalis Steiropoulos, Argyris Tzouvelekis, Evangelia Nena, Demosthenes Bouros

**Affiliations:** ^1^Department of Pneumonology, Medical School, Democritus University of Thrace, 68100 Alexandroupolis, Greece; ^2^Laboratory of Hygiene and Environmental Protection, Medical School, Democritus University of Thrace, 68100 Alexandroupolis, Greece

## Abstract

Interstitial lung diseases (ILDs) represent a heterogeneous group of more than two hundred diseases of either known or unknown etiology with different pathogenesis and prognosis. Lung cancer, which is the major cause of cancer death in the developed countries, is mainly attributed to cigarette smoking and exposure to inhaled carcinogens. Different studies suggest a link between ILDs and lung cancer, through different pathogenetic mechanisms, such as inflammation, coagulation, dysregulated apoptosis, focal hypoxia, activation, and accumulation of myofibroblasts as well as extracellular matrix accumulation. This paper reviews current evidence on the association between lung cancer and interstitial lung diseases such as idiopathic pulmonary fibrosis, sarcoidosis, systemic sclerosis, dermatomyositis/polymyositis, rheumatoid arthritis, systemic lupus erythematosus, and pneumoconiosis.

## 1. Introduction

Interstitial lung diseases (ILDs) represent a heterogeneous group of more than two hundred diseases of either known or unknown etiology with different pathogenesis and prognosis. It is estimated that one-third is attributed to endogenous and exogenous causes including environmental/occupational factors, infections, medications, radiation, and collagen diseases. The remaining two-thirds are characterized as idiopathic since no specific cause is recognized [1].

Lung cancer is the leading cause of cancer death in the developed countries with an incidence that is augmenting steadily worldwide, particularly among women [2]. Even though cigarette smoking and exposure to inhaled carcinogens are the major causes of lung cancer, a link between ILDs and lung cancer has been suggested. The first report was published in 1939 [3], and since then pulmonary fibrosis has been investigated for its role in tumor formation and development. Although different hypotheses have suggested various interpretations for this association, specific mechanisms have never been established. This paper will review the relationship between ILDs and lung cancer.

## 2. ILDs as a Cause of Lung Cancer

Several common features in the pathogenesis of ILDs and lung cancer have been determined during the last years. The continuous accumulation and the rapid proliferation of differentiated fibroblasts in the regions of repeated epithelial injury, in combination with an increased resistance in apoptosis, features of pulmonary fibrosis, represent pathogenic mechanisms similar to those followed by cancer cells [4], including unlimited cell multiplication, cellular immortality, and rapid immigration, which characterize cancer metastasis.

The increase in concentration of carcinoembryonic antigen (CEA) that has been detected in bronchoalveolar lavage fluid of patients with fibrosing alveolitis is associated with lung cancer [5]. This increase could reflect hyperplasia and metaplasia leading to the development of pulmonary carcinoma. However, high levels of CEA are also found in bronchoalveolar lavage fluid of smokers, and their measurement has restricted value in the diagnosis of malignancy [6].

Another feature of pulmonary fibrosis is epithelial-mesenchymal transition, a phenomenon according to which, type II epithelial alveolar cells are transformed into mesenchymal cells. These produce fibroblasts and myofibroblasts which contribute directly to the fibrotic event [7]. Studies have demonstrated that exposure of epithelial cells to matrix metalloproteinases may lead to increased levels of reactive oxygen species that promote this differentiation to myofibroblasts. Since the implication of defective matrix metalloproteinase expression and increased levels of reactive oxygen species are also characteristics of malignancy, a relationship between the two pathologies could be presumed [8]. In addition, microRNAs seem to participate to the epithelial-mesenchymal transition procedure, and the loss of specific microRNAs, such as let7d, is considered responsible of lung cancer development as well as pulmonary fibrosis induction [9, 10].

Moreover, the pathogenesis of idiopathic pulmonary fibrosis (IPF) still remains unclear. It is hypothesized that an unidentified stimulus produces repeated episodes of acute lung injury followed by a pathological wound healing [11]. This repeated epithelial injury could predispose to a series of genetic mutations including atypia, metaplasia, and dysplasia with final result the development of lung cancer. Previous studies demonstrated an augmented expression of p53 and p21 in hyperplastic bronchial and alveolar epithelial cells of lung tissues in patients with idiopathic pulmonary fibrosis. This result could suggest that p53 and p21 are upregulated in association with chronic DNA damage causing an arrest in G1 or apoptosis so that DNA repair may become possible [12]. A correlation between chronic DNA damage and repair and mutation of p53 could be speculated. Studies examining sputum and lung tissue of patients with idiopathic pulmonary fibrosis have shown microsatellite instability and loss of heterozygosity in genes that participate in apoptosis and cellular proliferation [13, 14]. Both processes have been previously reported in the development of lung carcinomas [15].

DNA hypomethylation is considered a hallmark of cancer [16]. Rabinovich et al. [17] analyzed global methylation patterns of IPF using CpG island microarrays, demonstrating altered methylation patterns compared to normal lung samples. In addition, IPF methylation pattern was much more similar to lung cancer, although tissue samples for lung cancer and controls were from the same patient. In particular, 402 CpG islands overlapped between IPF and lung cancer, representing a 65% of CpG islands that have altered methylation pattern in IPF lung samples. However, although hypomethylation was found to a great extent in IPF, changes are not as extensive as in cancer and do not involve global changes in long interspersed nuclear element 1 (LINE-1) methylation.

Moreover, telomerase deficiency and subsequent telomere shortening have been implicated in the pathogenesis of IPF. Similar changes are strongly associated with lung cancer, although their potential role is possibly different [18]. Mutations in TERT gene which encodes the catalytic component of telomerase and one heterozygous mutation in TERC which is the essential RNA component of telomerase were associated with increased susceptibility to developing IPF [19]. Another study demonstrated that two families developing pulmonary fibrosis shared two nonsynonymous substitutions in the TERT genes: V791I and V867M. The mutations were associated with telomere shortening leading to defects in the repeat addition processivity and contributing to the disease development [20]. Furthermore, 25% of individuals with sporadic IPF and without a mutation in TERT or TERC were found to exhibit telomere shortening in their circulating leukocytes [21]. Although telomerase role in lung cancer is not identical to that of IPF, its implication in the pathogenesis of both diseases raises suspicions for a potential common pathogenetic drive.

Based on the above results, Wang et al. identified 2 rare missense mutations in the gene encoding surfactant protein A2 (SPA2). Both mutations were predicted to disrupt protein structure and were present in 2 large families with IPF and adenocarcinoma of the lung [22]. Recombinant proteins carrying these mutations are not secreted from the endoplasmic reticulum. These data support the disturbance of protein trafficking due to the mutations, leading to the development of IPF and lung cancer.

## 3. Idiopathic Pulmonary Fibrosis and Lung Cancer

The association between IPF and lung cancer has been previously studied with various results, regarding the histologic type and the location of cancer in IPF patients. Nagai et al. [23] in a group of 99 patients with IPF reported lung cancer in 31 patients (31.3%) with a predilection for periphery and the lower lobes. The most common histologic type was squamous cell carcinoma (*n* = 14, 45.2%). The prevalence of cancer was not related to the radiologic stage of pulmonary fibrosis. Cigarette smokers with IPF were at a greater risk for developing lung cancer.

Park et al. [24] examined patients with combined IPF and lung cancer. The rate of presence of lung cancer within a fibrotic region was low (*n* = 23, 37%), and there was a preference for the periphery of the lung (*n* = 40, 56%) and the upper lobes (*n* = 33, 52%). An association between cigarette smoking and male sex was established, and the most common histologic type was squamous cell carcinoma.

Aubry et al. [25] compared three groups of patients: patients with IPF, patients with lung cancer and patients with both conditions. A major localization of cancer to the periphery in the fibrotic areas was described, and lower lobes were more frequently interested. The most frequent histological type was squamous cell carcinoma (16 cases out of 24). Males (male-to-female ratio 7 : 1) of older age (mean age 72.3 years) and cigarette smokers presented major predilection in developing IPF and lung cancer. Although the power of this study was limited due to the small size of the sample included, survival of patients with both IPF and lung cancer (2.3 years) seemed more similar to that of patients with carcinoma only (1.6 years) compared to that of single idiopathic pulmonary fibrosis.

Using a longitudinal computerized dataset to determine the incidence of cancer in patients with IPF, Le Jeune et al. [26] concluded that the incidence of cancer was higher in patients with IPF compared to the general population (rate ratio 1.51, 95% confidence interval 1.20–1.90). This finding was determined by an increase in the incidence of lung cancer (rate ratio 4.96, 95% confidence interval 3.00–8.18), while no increase in the risk for other types of cancers was found.

On the contrary, in a large analysis of mortality data in the United States (death certificates between 1979 and 1991) Wells and Mannino [27] found that lung cancer occurred less frequently among decedents with pulmonary fibrosis (4.8%) than in patients with obstructive pulmonary disease (10.06%) and asbestosis (26.6%) compared to the general population (6.48%). Limitation of this study is the possibility of underestimating the incidence of pulmonary fibrosis, due to its exclusion from the death certificates in addition to the difficulty in determining lung cancer rates for long-term survivors in comparison to short-term survivors with pulmonary fibrosis, since no data on the duration of the disease was provided [28].

The incidence and clinical characteristics of synchronous multiple lung cancer in patients with IPF was studied by Mizushima and Kobayashi [29]. Most of the lung cancer cases were observed in males and cigarette smokers with a predilection for the lower lobes and the peripheral regions of the lung. A high incidence of small-cell carcinoma was registered and the most common combinations were between small-cell carcinoma-squamous cell carcinoma (9 cases out of 23) and small-cell carcinoma-adenocarcinoma (7 cases out of 23).

Finally, the presence of finger clubbing is associated with the presence of both IPF and lung cancer (95%) than in IPF only (63%) and this phenomenon often preceded the clinical evidence of cancer [30].

**Figure 1:**
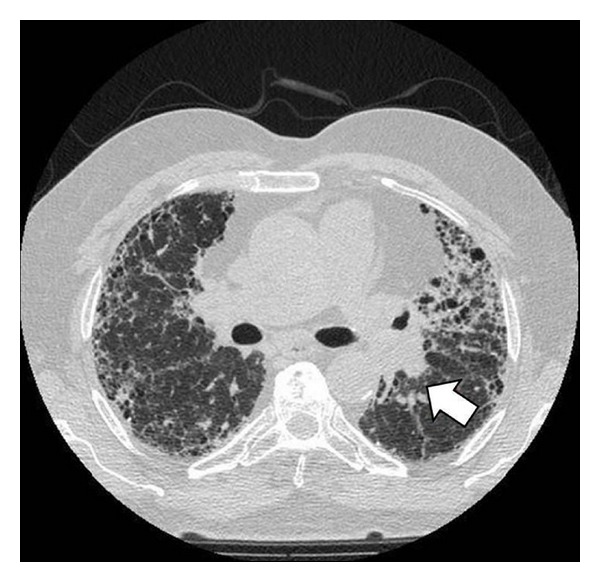
CT scan of a 69-years-old male with IPF and squamous cell carcinoma.

**Figure 2:**
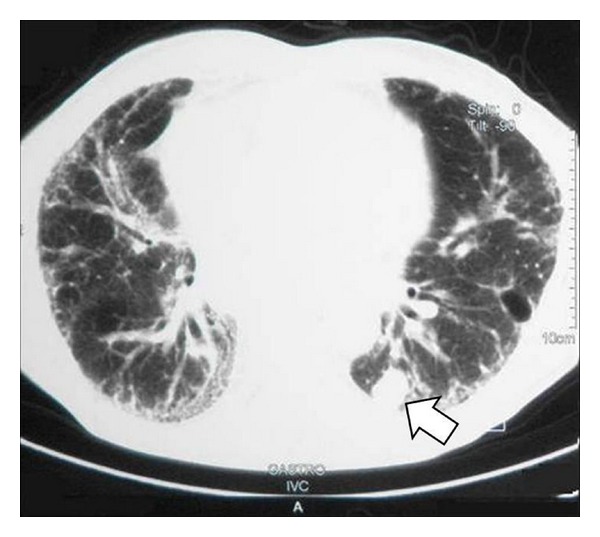
CT scan of a 72-years-old male with IPF and adenocarcinoma.

In conclusion, there is an evident association between lung cancer and IPF [28]. There is a predilection for male gender (Figures 1 and 2), cigarette smokers, and old age. Lower lobes and peripheral regions are more commonly involved.

## 4. Sarcoidosis and Lung Cancer

Sarcoidosis is an inflammatory granulomatous disease with its cause remaining largely unknown. Several speculations concerning the association between lung cancer and sarcoidosis have been made, and the conducted studies have reached to conflicting results. There are studies that display a positive association between sarcoidosis and lung cancer. Brincker and Wilbek [31] suggested that immunological deficiencies occurring in sarcoidosis could predispose to a higher incidence of cancer in sarcoidosis. In this study, lung cancer in patients with sarcoidosis developed three times more frequently in comparison to the general population. Yamaguchi et al. [32] in a cohort study of 1411 sarcoidotic patients, examined excess death due to lung cancer using standardized mortality rate. The standardized mortality rate for lung cancer was 3.26 (5.56 for males and 3.03 for females), indicating that sarcoidosis could be a risk factor for lung cancer.

In a cohort study of 474 sarcoidosis patients followed for a period of 24 years, the relative risk for lung cancer was doubled during the first decade of follow-up, but it was significantly decreased later [33]. In another cohort study which comprised 254 patients with sarcoidosis followed for a median time of 25 years, 5 cases of lung cancer (4 patients were smokers and 1 with unknown smoking history) were observed among 33 newly diagnosed different cases of cancer, with a standardized incidence rate of 2.0 (95% confidence interval 0.7–4.7) [34].

On the contrary, Boffetta et al. [35] found a decreased risk for lung cancer in a group of patients with sarcoidosis of different ethnic groups, in comparison to nonsarcoidotic patients of the same ethnic groups (relative risk 0.60 and 95% confidence interval 0.42–0.85). Another study from Denmark did not report an increased occurrence of malignant neoplasms, lung cancer included among 555 patients with sarcoidosis [36]. Finally, Marschke reported a limited occurrence of lung cancer in 2700 cases of sarcoidosis [37].

Limited data is provided in the above-mentioned studies regarding the significance of tobacco smoking in the development of lung cancer among sarcoidosis patients. Smoking has been related to decreased incidence and prevalence of sarcoidosis [38–40], even though later studies seem to argue this belief [41]. However, an inverse relationship between smoking habit and sarcoidosis could explain the low prevalence of lung cancer in sarcoidosis patients found in several studies.

In conclusion, evidence regarding the correlation between lung cancer and sarcoidosis seems to be inconclusive. In accordance with an augmented risk for cancer seen in other inflammatory respiratory disorders [30], parenchymal involvement during disease progression may transiently expose sarcoidosis patients to increased risk for lung cancer. 

Lung cancer usually develops several years after the diagnosis of sarcoidosis, a fact that may reflect the increased cancer rate in older people [28], even though in some cases both diseases are simultaneously presented [31, 42].

## 5. Systemic Sclerosis and Lung Cancer

The first reported association between lung cancer and systemic sclerosis (SSc) was published nearly 50 years ago, in a case report of alveolar carcinoma [43]. Since then, the correlation between the two diseases has been examined in several studies [44–46].

In a study that included 112 Korean patients with SSc, 4 cases of lung cancer were recognized [47]. All patients were female, nonsmokers, and the histological type was non-small cell lung cancer. There were no significant differences between patients who developed lung cancer and those with SSc only in their previous treatment, autoantibodies, smoking habits, and lung involvement.

A population based cohort study [48] with 441 patients with SSc reported that the standardized incidence rate for lung cancer was 5.9 (95% confidence interval 3.05–10.31). In the same study, the standardized incidence rate for all cancer types was also increased (1.99, 95% confidence interval 1.46–2.65).

In a study conducted by Chatterjee et al. [49] in the Detroit metropolitan area, which determined the incidence of all types of cancer in patients with SSc, 538 patients were selected using a match between a registry for scleroderma patients and a cancer database. In 45 patients, the diagnosis of cancer was set, and lung cancer was found to be the most common (10 cases out of 45). However, the incidence of lung cancer was not different from that of the general population in the Detroit area. The authors suggested that local cancer rates should be taken into consideration when different SSc cohorts are compared.

Adžić et al. [50] studied 375 patients with connective tissue diseases. Lung cancer was identified in 24 patients, out of whom 11 patients were affected by SSc (46%). Kanaji et al. reported a link between small-cell lung cancer and SSc-associated ILD [51]. Hesselstrand et al. [52] analyzed survival rates, cause of death, and occurrence of fatal malignant neoplasms in a population of 249 patients with SSc. Out of 49 deaths, 24 were caused by pulmonary complications and 7 were attributed to lung cancer. The most common histologic type was adenocarcinoma (5 out of 7). In this study, cyclophosphamide treatment was not associated with an increased incidence of fatal malignancies.

In another report, 917 patients with SSc were studied. Risks were elevated for lung cancer with a standardized incidence rate of 4.9 (95% confidence interval 2.8–8.1). Lung cancer followed the diagnosis of scleroderma after 5 to 9 years and the most common histologic types were adenocarcinoma and squamous cell type [53]. Winkelmann et al. [54] found 14 cases of lung cancer in a population of 3550 patients with SSc. The diagnosis of lung cancer came after that of SSc by 6 years in 8 cases and the most common histologic type was small-cell lung cancer (5 out of 14 cases). Interestingly, 9 out of 14 patients who developed lung cancer were nonsmokers.

In a retrospective study of 248 patients with SSc, lung cancer was found in 7 patients, and an association with pulmonary fibrosis was registered [55]. Roumm and Medsger [56] studied a group of 262 patients with SSc and detected 14 malignancies (5%). An increased incidence of lung cancer was observed, which occurred in the setting of pulmonary fibrosis. Interestingly, no association between lung cancer insurgence and cigarette smoking was noted. In another study with 123 scleroderma patients, 3 cases of lung cancer were registered, and the association between CREST syndrome and anticentromere antibodies was noted [57].

In a nested case control study [58], smokers patients with SSc had a 7-fold risk to develop lung cancer, compared to nonsmokers (*P* = 0.008). In the same study pulmonary fibrosis and antitopoisomerase antibodies did not seem to increase the risk of lung cancer and peripheral lung tumors occurred earlier in the course of the disease than bronchogenic tumors (*P* = 0.05). 

The pathogenetic link between SSc and lung cancer is possibly the inflammatory and fibrotic environment combined with immunologic abnormalities noted in these patients, which may predispose to cancer development [28]. The extended use of immunosuppressive agents has also been suggested as a probable cause of lung cancer development [48].

## 6. Dermatomyositis/Polymyositis and Lung Cancer

Dermatomyositis/polymyositis (DM/PM) has been associated with lung cancer in different studies. It has been reported that, in some cases, myositis follows the clinical course of cancer; supporting the hypothesis that myositis could be a paraneoplastic disorder [59, 60].

In a population-based study conducted in Taiwan [61] that included 1059 patients with DM and 661 patients with PM, patients with DM presented a 10-fold increased risk for cancer with a 31-fold increased risk for lung cancer compared to the general population. In this study, most malignancies were diagnosed shortly after the diagnosis of DM/PM, and younger patients were at higher risk for malignancies.

In a retrospective study of 115 cases of DM associated with malignancy [62], lung cancer accounted for 17.4% (20 out of 115 cases). In the same study, cancer was reported mainly in patients older than 40 years, and was diagnosed usually within the first year after the onset of DM.

Hill et al. [63], in a population-based study, identified 198 patients with DM and 137 patients with PM both associated with cancer. Patients with DM and PM presented an increased risk for lung cancer (standardized incidence rate 5.9 and 2.8, resp.). In both groups, cancer was diagnosed mostly after the diagnosis of myositis.

In another study, development of DM was more frequently associated with the development of cancer, and the more frequent localizations were breast, lung, pancreas, and colon cancers. Cancer was identified 1 year after the diagnosis of DM in 87.5% of the cases and mainly males of age above 45 years. Interestingly, in patients with DM and cancer, a weaker association with the presence of ILD was revealed [64].

The presence of DM/PM was associated with all histological types of lung cancer, including small-cell lung cancer, squamous cell carcinoma, and adenocarcinoma [65]. Cancer incidence remains increased during the first two years after the diagnosis of DM/PM, but had no significant excess afterwards [66].

The specific pathogenetic mechanism between DM/PM and lung cancer development still remains undetermined. Different hypotheses suggest a common environmental or genetic cause, or malignancy associated to the immunosuppressive therapy of the disease [28].

## 7. Rheumatoid Arthritis and Lung Cancer

Rheumatoid arthritis (RA) is related to an increased risk of hematologic malignancies, mainly lymphomas [67, 68]. Nevertheless, an association between RA and lung cancer has also been described.

In an observational cohort study [67] that included 7566 patients with RA followed up for four years, an increased risk for lung cancer was established with a standardized incidence rate of 2.29 (95% confidence interval 1.57–3.21). Male gender and older age were identified as risk factors for malignancy. Another retrospective cohort study also found an increased risk for lung cancer among males with RA [68].

In the study of Khurana et al. [69], patients with RA presented a 43% probability of encountering lung cancer compared to those without the disease (odds ratio 1.43), revealing a significant association between the two diseases for the population studied. Takayanagi et al. [70] investigated the relationship of usual interstitial pneumonia and smoking in patients with RA and lung cancer by studying a group of 86 patients. Out of 86 patients, 28 had lung cancer, and 14 out of them presented with usual interstitial pneumonia (50%). In the group of both RA and usual interstitial pneumonia that accounted 72 patients, 14 presented lung cancer (19.4%).

A meta-analysis examining the incidence of malignancy in RA patients demonstrated an increased risk of lung cancer with a standardized incidence rate of 1.63 (95% confidence interval 1.43–1.87) [71]. In a cohort study of 789 patients with RA, published at the same year, an increased risk for leukemia, non-Hodgkin lymphoma, and lung cancer was reported. Male gender, age, and the use of any cytotoxic drugs apart from methotrexate were identified as predictive factors for the development of cancer [72]. In a previously conducted study, lung cancer risk among patients with RA was increased, with a standardized incidence rate of 1.32 for males (95% confidence interval 1.2–1.5) and 1.44 for females (95% confidence interval 1.3–1.6) [73]. Two older publications from Denmark also demonstrated a positive correlation between RA and lung cancer in a large population of 20.000 lung cancer patients [74, 75].

There is no known pathogenic mechanism that associates RA and lung cancer. Speculations include a persistent immunologic stimulation induced by the disease itself and the use of cytotoxic agents as treatment for RA [28].

## 8. Systemic Lupus Erythematosus and Lung Cancer

Systemic lupus erythematosus (SLE) is a chronic, multisystem, and autoimmune disease that primarily affects women. Even though morbidity and mortality have improved, data confirm an increased risk for all cancers compared to the general population. This is primarily caused because of an augmented incidence of non-Hodgkin lymphoma and Hodgkin lymphoma, but the risk of developing lung cancer in patients with SLE seems to be increased as well [76].

In a study, on 30 cases of lung cancer in patients with SLE, the most common histological type was adenocarcinoma (8 cases) followed by small-cell lung cancer (6 cases) and squamous cell carcinoma (6 cases). Most patients were female (75%), smokers (71%), and only a small percentage underwent therapy with immunosuppressive agents (20%) [77].

A multicenter cohort study (23 centers from different countries) found an increased risk for all cancers (standardized incidence rate 1.15, 95% confidence interval 1.05–1.27), including hematologic malignancies and lung cancer (standardized incidence rate 1.37, 95% confidence interval 1.05–1.76). For all cancers, the risk seemed higher for patients with early SLE, even though the majority of cancer occurred at least 1 year after the diagnosis of SLE. Female gender was more influenced in accordance to the fact that SLE is a female's disease [78].

In a cohort of 238 unselected patients with SLE [79], lung cancer was identified in 36 patients (32 women and 4 men). An increased risk for lung cancer was reported (odds ratio of 1.72), but with a confidence interval ranging between 0.36–4.95 (*P* = 0.254). Females were at a higher risk of developing cancer than men (odds ratio 1.45 versus 1.03), respectively. The mean age of diagnosis for malignancy was 62.7 years (range 43–86), and for most patients (27 out of 36) the diagnosis of malignancy came after that of SLE. In another cohort of 5715 SLE patients, 443 malignancies were recognized and lymphomas represented the major excess risk. The risk of lung cancer was also increased with a standardized incidence rate of 1.73 (95% confidence interval 1.25–2.32) [80]. 

In a cohort study of 616 women with SLE, 30 cases of malignancy were documented. Lung cancer was the only cancer increased in all women with a standardized incidence rate of 3.1 (95% confidence interval 1.3–7.9) [81]. Mellemkjaer et al. [82] in a cohort of 1585 patients with SLE found an excess of non-Hodgkin lymphoma and an increased risk of developing lung cancer with a relative risk of 1.9. 

The mechanism that associates SLE and lung cancer remains undetermined. A hypothesis suggests that genetic susceptibility could predispose to the development of both SLE and lung cancer and the development of the former may also be facilitated by smoking [83]. Another theory associates lung cancer and SLE, through the development of fibrotic lung disease, suggesting that exposure to chronic inflammation due to pneumonitis could induce DNA damage that may lead to lung cancer [84]. Medication exposure could also have a significant role in the development of cancer, but the role of immunosuppressive therapy is yet to be established [85].

## 9. Pneumoconiosis and Lung Cancer

Occupational exposure to dust can cause a series of diseases with different clinical and radiological patterns called pneumoconiosis. *Silicosis* is a disease caused by the exposure to silica, a material used in a large number of industries including mines and quarries, production of granite, ceramics, pottery, and steel. The role of silicosis as a risk factor for lung cancer has been considered in many studies but still remains uncertain, mainly because of the difficulty to exclude cigarette smoking as an additional risk factor [86, 87].

In a study conducted to establish the risk of lung cancer among uranium miners in France, a significant association between lung cancer and silicosis was revealed (odds ratio for silicosis 3.6, 95% confidence interval 1.4–8.9) [88]. A case control study in an area of Japan with a high prevalence of silicosis concluded that an increased mortality by lung cancer could be associated to silica exposure. The most common histologic type was small-cell lung cancer [89]. Finkelstein in his review suggested that an exposure to silica may increase the lifetime risk of developing silicosis and lung cancer [90].

Three studies which examined industrial sand workers pointed out the relation between silica exposure, silicosis and lung cancer [91–93]. The standarized mortality rate for lung cancer was 1.60 (with 95% confidence interval of 1.31–1.93) in one study [92], while in two of these studies lung cancer risk seemed related to average silica concentration and cumulative exposure, but not to length of employment [91, 93].

In a retrospective study of 14929 workers compensated for silicosis in Italy, an increased mortality due to lung cancer particularly among males was established [94]. In a similar Australian study, the excess of mortality due to lung cancer among silicotic patients was confirmed (standardized mortality rate for lung cancer 1.9, 95% confidence interval 1.5–2.3), even though the presence of chronic obstructive pulmonary disease and cigarette smoking could have influenced the results [95].

In a meta-analysis, silicosis increased the risk for lung cancer (relative risk 2.37, 95% confidence interval 1.98–2.84). The same study reported an increased risk of lung cancer among silicotic patients due to cigarette smoking (relative risk 4.47, 95% confidence interval 3.17–6.30) [96]. Another meta-analysis that included 31 studies reported a common standardized mortality rate of 2.45 (95% confidence interval 1.63–3.66) without an adjustment for smoking, and a standardized mortality rate of 1.60 (95% confidence interval 1.33–1.93) when data was adjusted for smoking. In the same study, authors concluded that lung cancer risk among silicosis patients could be overestimated in the current literature due to biases inherent to observational studies [97]. In a review of the epidemiological studies conducted between 1996 and 2005, the relative risk for lung cancer in cohort studies in patients with silicosis was 1.69, and the relative risk in case control studies was 3.27 [98].

Several hypotheses about the causal effect of silica in lung cancer have been suggested. There is evidence of immunological dysregulation caused by silica that could lead to immunity alterations [99, 100]. Silica is involved in the production of reactive oxygen species and induces oxidative stress in bronchial epithelial cells, while alpha-quartz exposure produced cytotoxic effects and DNA damage in lung epithelial cells, all processes involved in carcinogenesis [101, 102]. Studies demonstrating mutations of the p53 gene [103, 104] and an aberrant promoter methylation of tumor suppressor genes [105] induced by silica exposure indicates an action in a molecular level. Silica can also act indirectly by favoring the absorption of other carcinogens like polyaromatic hydrocarbons contained in cigarette smoke, a process facilitated by an additional impairment of the pulmonary clearance [28].

*Asbestosis* is another ILD that develops after the exposure to asbestos fibers. The causality between exposure to asbestos and the induction of lung cancer and mesothelioma is well known and proven by many studies [106–110]. There is difficulty in determining the exact epidemiology of asbestos-induced lung cancer because most cases cannot be easily distinguished from those attributable to other causes. However, asbestos presents a carcinogenic effect even in nonsmokers and may contribute to the induction of lung cancer in smoking patients [111].

An association between the dimension of asbestos fibers and lung disease has been established. In a study examining the role of the dimension of chrysotile asbestos fibers as a determinant of lung disease [112], both lung cancer and asbestosis were associated with exposure to thinner fibers (<0.20 *μ*m). Exposure to longer fibers (>10 *μ*m) was strongly associated with the development of lung cancer, but no relation between fiber length and asbestosis was observed. 

An association between the degree of exposure to asbestos and lung cancer was also reported. In a case control study of 1139 asbestos workers [113], the odds ratio for lung cancer in the high exposure group was 3.66 (95% confidence interval 1.61–8.29) compared to 1.25 for the medium exposure group. In the same study, the risk for lung cancer was further elevated between smokers. Similar results are presented in a follow-up study in a cohort of 3072 chrysotile textile workers [114].

The majority of epidemiological studies reported an almost linear relationship between asbestos dose and lung cancer risk. There is no clear evidence of a correlation between the time of first exposure and lung cancer development. Latency differs between cases from 5 to 9 years after the first exposure and rises until 30 years [115]. Lung cancer induced by asbestos exposure includes all histological types and presents a predilection for the main bronchi, even though smaller bronchi and peripheral lung regions could also be involved [116].

The exact mechanism by which asbestos induces lung cancer is still under investigation. Studies revealed that asbestos could be a tumor promoter and that is implicated in the later stages of carcinogenesis of lung cancer [117]. The carcinogenic activity of asbestos also includes free radicals production, since a large quantity of free radicals is produced by the phagocytic cells once they are filled with asbestos fibers due to their incapacity of digesting those fibers [118]. There is also evidence indicating that asbestos fibers could act as concentrators of carcinogenic molecules including the components of cigarette smoke [118]. Another theory postulates that asbestos may have an effect on chromosomes during cell division which may lead to genetic alterations such as activation of oncogenes or inactivation of tumor suppressor genes [118]. Finally, asbestos produces immunological alterations by inducing an impairment of cytotoxicity of natural killer cells and by modifying the expression of natural killer cell-activating receptors [119]. 

The spectrum of pneumoconiosis has recently expanded with the development of pneumoconiosis due to novel exposures [120]. The potential associations between these emerging occupational ILDs and lung cancer is an issue that will certainly become of interest in the next few years.

## 10. Conclusion

Evidence suggests an association between ILDs and lung cancer development. Characteristics of ILDs (accumulation and the rapid proliferation of differentiated cells and increased resistance in apoptosis) are similar to those of cancer metastases (unlimited cell multiplication, cellular immortality, and rapid immigration).

Epidemiological evidence varies, due to the different study designs and the different study populations (Table 1). The pathogenetic mechanism of this association is difficult to interpret mainly due to the different manifestations of each disease.

## Figures and Tables

**Table 1:** Summary of the studies on incidence of lung cancer in interstitial lung diseases.

Study	Patients	Incidence of lung cancer
	IPF	
Nagai et al. [23]	99	31 (31.3%)
Park et al. [24]	281	63 (22.4%)
Le Jeune et al. [26]	1064	29 (2.7%)
	Sarcoidosis	
Le Jeune et al. [26]	1153	4 (0.3%)
Seersholm et al. [34]	254	5 (1.9%)
Boffetta et al. [35]	5768	37 (0.6%)
Romer et al. [36]	555	1 (0.2%)
	Scleroderma	
Kang et al. [47]	112	4 (3.5%)
Hill et al. [48]	441	12 (2.7%)
Chatterjee et al. [49]	538	10 (1.8%)
Abu-Shakra et al. [55]	248	7 (2.8%)
Kyndt et al. [57]	123	3 (2.4%)
	DM/PM	
Huang et al. [61]	1720	30 (1.7%)
Zhang et al. [62]	115	20 (17.4%) DM
Hill et al. [63]	1532	39 (2.5%)
	RA	
Yamada et al. [67]	7566	34 (0.4%)
Parikh-Patel et al. [68]	84475	762 (0.9%)
Khurana et al. [69]	9015	247 (2.7%)
Takayanagi et al. [70]	86	14 (16.2%)
Abásolo et al. [72]	789	6 (0.7%)
	SLE	
Bin et al. [77]	9500	30 (0.3%)
Bernatsky et al. [78]	9547	62 (0.6%)
Ramsey-Goldman et al. [81]	616	4 (0.6%)

Abbreviations: DM: dermatomyositis, IPF: idiopathic pulmonary fibrosis, PM: polymyositis, RA: rheumatoid arthritis, SLE: systemic lupus erythematosus.

## Abbreviations

CEA: Carcinoembryonic antigen
DM: Dermatomyositis
ILDs: Interstitial lung diseases
IPF: Idiopathic pulmonary fibrosis
PM: Polymyositis
RA: Rheumatoid arthritis
SLE: Systemic lupus erythematosus
SSc: Systemic sclerosis.
